# ﻿Molecular phylogeny and morphology reveal three new plant pathogenic fungi species (Septobasidiales, Basidiomycota) from China

**DOI:** 10.3897/mycokeys.111.125933

**Published:** 2024-12-30

**Authors:** Qianquan Jiang, Zhengli Kang, Xubo Wang, Changlin Zhao

**Affiliations:** 1 Yunnan Provincial Key Laboratory for Conservation and Utilization of In-forest Resource, the Key Laboratory of Forest Resources Conservation and Utilization in the Southwest Mountains of China Ministry of Education, Southwest Forestry University, Kunming 650224, China Southwest Forestry University Kunming China; 2 College of Forestry, Southwest Forestry University, Kunming 650224, China Southwest Forestry University Kunming China

**Keywords:** Forest disease, phylogenetic analyses, taxonomy, wood-inhabiting fungi, Yunnan Province

## Abstract

Three new fungal species, *Septobasidiummacrobasidium*, *S.puerense* and *S.wuliangshanense*, are proposed based on a combination of the morphological features and molecular evidence. The taxon *S.macrobasidium* is characterized by the coriaceous basidiomata with a cream surface, cylindrical basidia, straight, 4-celled, subglobose or ovoid probasidia and thin-walled, narrowly cylindrical basidiospores with septa, measuring as 7–9 × 3.5–4.5 µm, the haustoria consisting of irregularly coiled hyphae; in addition, this fungus was found associated with the insect of Diaspididae. The species *S.puerense* is characterised by resupinate coriaceous basidiomata with a cinnamon brown to chestnut brown surface, cylindrical or slightly irregular basidia, 2-3-celled, slightly curved, subglobose to pyriform probasidia, probasidia cell persistent after the formation of the basidia and the haustoria with two types consisting of irregularly coiled hyphae and spindle-shape. The fungus was found associated with the insect species *Pseudaulacaspispentagona*. The species *S.wuliangshanense* is characterised by the coriaceous basidiomata with a slightly brown surface, cylindrical or slightly irregular basidia, 2-3-celled, straight or slightly curved, pyriform, subglobose or ovoid and probasidia, haustoria consisting of irregularly coiled hyphae, associated with the insect genus *Aulacaspis*. Sequences of internal transcribed spacer region (ITS) were analysed maximum likelihood, maximum parsimony and Bayesian inference methods. The new species *S.macrobasidium* was clustered with *S.maesae*. Furthermore, *S.puerense* was retrieved as a sister to *S.carestianum*. The phylogenetic tree, inferred from the ITS sequences, highlighted that *S.wuliangshanense* was the sister to *S.aquilariae* with strong supports. Application of PHI test to the ITS tree-locus sequences revealed no recombination level within phylogenetically related species.

## ﻿Introduction

Fungi are a diverse, monophyletic group of eukaryotes and these organisms show immense ecological and economic impacts for playing an important role in the ecosystems as diverse as soil, trees, with hidden layers within their substrate (James et al. 2020). Approximately 150 thousand species of fungi have been described (Cui et al. 2019; Dai et al. 2021; Wang and Zhao 2021; Sun et al. 2022; Liu et al. 2023). But the potential biodiversity of the group is likely to be 2.2–3.8 millions of species (Blackwell 2011; Taylor et al. 2014; Hibbett 2016; Dong et al. 2023). The diversity for flora of seed plants in Yunnan Province is higher than that in other areas of China. The number of discovered new fungal species totalled 1,345 from this province from 2000 to 2020 years. Endemic woody plants are rich in Yunnan, supplying rich and varied substrates for wood-decaying fungi. The pathogenic fungi with industrial, medicinal and economic value, comprise most basidiomycetes and ascomycetes growing on various kinds of living trees (Dai et al. 2015, 2021; Vinay et al. 2015; Wu et al. 2019; Luo et al. 2022).

Mutualistic and parasitic symbioses between fungi and plants are widely acknowledged to have profound influences on the evolution and ecology of terrestrial life, but less well-known are the symbioses between fungi and insects (Henk and Vilgalys 2007). The insects are protected from their enemies by the fungus and the fungus draws its nutriment from, and is distributed by, the insects. Under natural conditions, whereas the fungus cannot live without the insects, the insects are able to live without the fungus, but the life of insects unprotected by the fungus is precarious (Couch 1938).

Basidiomycete fungi have evolved many symbiotic associations with plants and animals, but fungi in the order Septobasidiales are the only large group of basidiomycetes that are obligately parasitic on insects. Understanding the evolution of insect parasitism and switches from plant parasitism in the Basidiomycota requires a phylogeny to place the order Septobasidiales within the Pucciniomycotina and to determine whether the different forms of insect parasitism in the order Septobasidiales and *Septobasidium* Pat. have a single origin. *Septobasidium* is a type of fungi that has a mutualistic relationship with insects. Although *Septobasidium* sterilize the individuals they parasitize, the fungi may protect uninfected individuals and thereby benefit the population of scale insects (Couch 1938). All fungi in the order Septobasidiales do not display this type of symbiosis. Some may be wholly parasitic because they do not form substantial protective structures. This fungus-insect symbiosis is important because of its unique altruistic and parasitic characteristics and its phylogenetic position within the Basidiomycota (Henk and Vilgalys 2007).

*Septobasidium* (Septobasidiaceae, Septobasidiales), erected by Patouillard (1892), typed by *Septobasidiumvelutinum* Pat., which is a large cosmopolitan genus characterised by perennial colonies on the surfaces of plant structures with colonies of scale insects, the basidiomata are usually white to cream, yellowish brown or brown hymenophore, rarely more brightly coloured, normally resupinate and felty in texture; their surfaces may be smooth, warty, or spiny (Patouillard 1892; Couch 1929); a monomitic hyphal system with simple septa, with or without probasidia, 2–4 celled cylindrical, curved, or straight basidia, basidiospores that are hyaline, thin-walled, smooth and cylindrical or fusiform and haustoria consisting of coiled or spindle-shaped hyphae (Ma et al. 2019). In a previous study, Couch (1938) divided it primarily based upon morphological characters related to the structure of the basidia and probasidia. Basidia may be curved or straight and may have one, two, three, or four cells. The probasidia either remains as an empty cell at the base of the mature basidia (persistent) or becomes a spore-bearing cell in the mature basidia (not persistent). Other characters used as indicators of major groups in *Septobasidium* include the layered nature of the thallus and the presence of pillar structures (Oberwinkler 1989). *Septobasidium* occurs on living leaves, stems and branches of a great variety of perennial plants, including gymnosperms, monocots and dicots. Inexperienced collectors may mistake them for corticioid basidiomycetes or even lichens (Couch 1929, 1935, 1938, 1946).

As with most basidiomycetes, basidia are produced so that they project toward the ground. Thus, the resupinate basidiomata often are found on the lower sides of branches. They occur on living rather than dead plant parts and away from the extreme tips of branches which distinguishes them from some resupinate species of Aphyllophorales (Couch 1938; Henk and Vilgalys 2007). About 305 species have been accepted into the genus worldwide (Patouillard 1892; Bresadola and Saccardo 1897; Burt 1916; Lloyd 1919; Couch 1929, 1935, 1938, 1946; Yamamoto 1956; Gómez and Henk 2004; Henk 2005; Lu and Guo 2009a, 2009b, 2010a, 2010b, 2011; Chen and Guo 2011a, 2011b, 2011c, 2012; Lu et al. 2010; Li and Guo 2013, 2014; Ma et al. 2019).

Molecular systematics has played a powerful role in inferring phylogenies within fungal groups since the early 1990s (White et al. 1990; Binder et al. 2013; Dai et al. 2015; Choi and Kim 2017). Classification of the kingdom of fungi has been updated continuously, based on the frequent inclusion of data from DNA sequences in many phylogenetic studies (Wijayawardene et al. 2020). However, molecular studies involving *Septobasidium* are rare (Henk and Vilgalys 2007; Zhao et al. 2017). One phylogenetic study of a single origin of insect symbiosis in the class Pucciniomycetes suggested that there is little or no support for *Septobasidium* as a monophyletic group (Henk and Vilgalys 2007). The previous study introduced a six-gene phylogenetic overview of Basidiomycota and allied phyla and confirmed that *S.carestianum* Bres. nested within the order Septobasidiales and grouped with *Helicobasidiummompa* Nobuj. Tanaka and *Thanatophytumcrocorum* (Pers.) Nees (Zhao et al. 2017).

During investigations into the wood-inhabiting fungi in Pu’er, Yunnan of China, samples representing three additional species belonging to genus *Septobasidium* were collected. Three new *Septobasidium* taxa were found that could not be assigned to any described species. To clarify the placement and relationships of these specimens, we carried out a phylogenetic and taxonomic study based on the ITS sequences. A description, illustrations, and phylogenetic analysis results of the new species are provided.

## ﻿Materials and methods

### ﻿Sample collection and herbarium specimen preparation

Fresh fruiting bodies of fungi growing on angiosperm branches were collected from Pu’er of Yunnan Province, P.R. China. The samples were photographed in situ and fresh macroscopic details were recorded. Photographs were recorded using a Jianeng 80D camera (Tokyo, Japan). All of the photos were stacked and merged using Helicon Focus Pro 7.7.5 software. Specimens were dried in an electric food dehydrator at 40 °C and then sealed and stored in an envelope bag and deposited in the herbarium of the Southwest Forestry University (SWFC), Kunming, Yunnan Province, P.R. China.

### ﻿Morphology

Macromorphological descriptions are based on field notes and photos captured in the field and lab. Color terminology follows Petersen (1996). Micromorphological data were obtained from the dried specimens when observed under a light microscope following the previous study (Ma et al. 2019). The following abbreviations are used: L = mean spore length (arithmetic average for all spores), W = mean spore width (arithmetic average for all spores), Q = variation in the L/W ratios between the specimens studied and n = a/b (number of spores (a) measured from given number (b) of specimens).

### ﻿DNA extraction and sequencing

Genomic DNA was obtained from dried specimens using the EZNA HP Fungal DNA Kit, according to the manufacturer’s instructions with some modifications. A small piece of dried fungal specimen (about 30 mg) was ground to powder with liquid nitrogen. The powder was transferred to a 1.5 ml centrifuge tube, suspended in 0.4 ml of lysis buffer and incubated in a 65 °C water bath for 60 min. After that, 0.4 ml phenol-chloroform (24: 1) was added to each tube and the suspension was shaken vigorously. After centrifugation at 13,000 rpm for 5 min, 0.3 ml supernatant was transferred to a new tube and mixed with 0.45 ml binding buffer. The mixture was then transferred to an adsorbing column (AC) for centrifugation at 13,000 rpm for 0.5 min. Then, 0.5 ml inhibitor removal fluid was added in AC for a centrifugation at 12,000 rpm for 0.5 min. After washing twice with 0.5 ml washing buffer, the AC was transferred to a clean centrifuge tube and 100 ml elution buffer was added to the middle of adsorbed film to elute the genomic DNA. ITS region was amplified with primer pairs ITS5 and ITS4 (White et al. 1990). The PCR procedure was as follows: initial denaturation at 95 °C for 3 min; followed by 35 cycles of 94 °C for 40 s, 58 °C for 45 s and 72 °C for 1 min; and a final extension of 72 °C for 10 min. The PCR products were purified and directly sequenced at barium Tsingke Biological Technology Limited Company. All newly generated sequences were deposited at GenBank (https://www.ncbi.nlm.nih.gov/genbank/) (Table 1).

**Table 1.** List of species, specimens and GenBank accession numbers of sequences used in this study. [* Indicates type materials].

Species	Specimen No.	GenBank accession No.	Preference
		ITS	
*Helicobasidiummompa*	DAH h1	DQ241472	Henk and Vilgalys 2007
*Pachnocybeferruginea*	DAH pf1	DQ241473	Henk and Vilgalys 2007
*Septobasidiumalni*	DAH FP3	DQ241441	Henk and Vilgalys 2007
*S.aquilariae*	CLZhao 6610	MK804520	Ma et al. 2019
*S.aquilariae*	CLZhao 6611	MK804521	Ma et al. 2019
*S.aquilariae*	CLZhao 6612	MK804522	Ma et al. 2019
*S.aquilariae*	CLZhao 6613	MK804523	Ma et al. 2019
*S.aquilariae*	CLZhao 6614	MK804524	Ma et al. 2019
*S.arachnoideum*	DAH 025	DQ241443	Henk and Vilgalys 2007
*S.bogoriense*	998434	HM209414	Lee et al. 2023
*S.broussonetiae*	998436	HM209416	Unpublished
*S.burtii*	DAH 062	DQ241444	Henk and Vilgalys 2007
*S.canescens*	DAH 323	DQ241446	Henk and Vilgalys 2007
*S.carestianum*	DJM 644	DQ241448	Henk and Vilgalys 2007
*S.castaneum*	DAH 052	DQ241447	Henk and Vilgalys 2007
*S.cavarae*	DJM FP1	DQ241445	Henk and Vilgalys 2007
*S.fumigatum*	DAH 005	DQ241451	Henk and Vilgalys 2007
*S.gomezii*	DAH 031	DQ241462	Henk and Vilgalys 2007
*S.grandisporum*	DAH 065	DQ241453	Henk and Vilgalys 2007
*S.griseum*	DAH 016	DQ241454	Henk and Vilgalys 2007
*S.hainanense*	998437	HM209417	Lee et al. 2023
*S.kameii*	998432	HM209412	Lee et al. 2023
***S.macrobasidium***	CLZhao 9624*	**PP532758**	**Present study**
***S.macrobasidium***	**CLZhao 9658**	**PP532759**	**Present study**
*S.maesae*	998433	HM209413	Unpublished
*S.marianiae*	LJF 7006	MK809161	Henk and Vilgalys 2007
*S.marianiae*	DAH 283b	DQ241456	Henk and Vilgalys 2007
*S.michelianum*	DAH FP5	DQ241457	Henk and Vilgalys 2007
*S.pallidum*	998435	HM209415	Unpublished
*S.pilosum*	DAH 020	DQ241458	Henk and Vilgalys 2007
*S.pinicola*	DAH 013	DQ241459	Henk and Vilgalys 2007
*S.pseudopedicellatum*	DAH 044	DQ241460	Lee et al. 2023
***S.puerense***	CLZhao 9430*	**PP532760**	**Present study**
***S.puerense***	**CLZhao 4298**	**PP532761**	**Present study**
*S.ramorum*	DAH 045a	DQ241450	Lee et al. 2023
*S.septobasidioides*	DAH 032	DQ241461	Henk and Vilgalys 2007
*S.sinuosum*	DAH 036	DQ241464	Henk and Vilgalys 2007
*S.taxodii*	DAH 194C	DQ241466	Henk and Vilgalys 2007
*S.velutinum*	DAH 024	DQ241467	Lee et al. 2023
*S.westonii*	DAH FP2001	DQ241468	Henk and Vilgalys 2007
*S.wilsonianum*	DAH 037	DQ241469	Henk and Vilgalys 2007
***S.wuliangshanense***	CLZhao 5809*	**PP532756**	**Present study**
***S.wuliangshanense***	**CLZhao 3666**	**PP532757**	**Present study**

### ﻿Phylogenetic analyses

**Figure 1.**
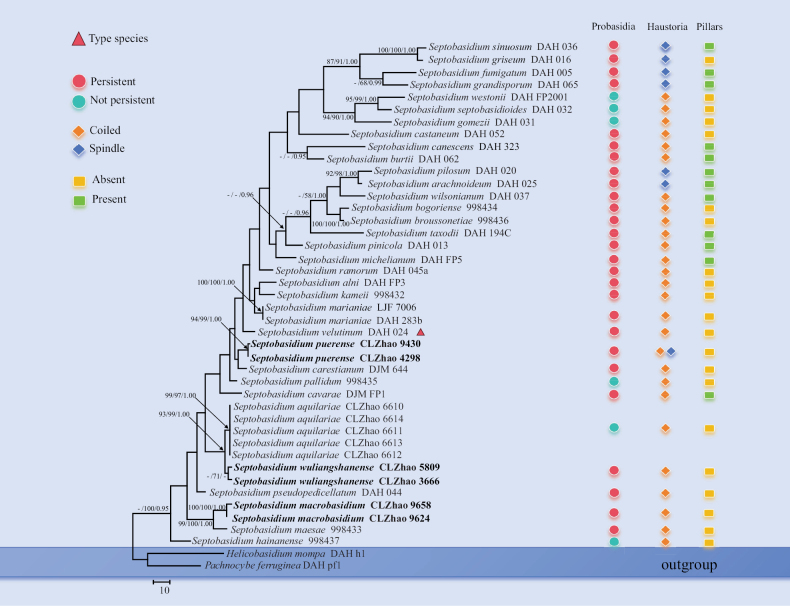
Maximum parsimony strict consensus tree illustrating the phylogeny of the three new species and related species in *Septobasidium*. Branches are labelled with maximum likelihood bootstrap values ≥ 70%, parsimony bootstrap values ≥ 50% and Bayesian posterior probabilities ≥ 0.95, respectively.

The sequences were aligned in MAFFT version 7 using the G-INS-i strategy (Katoh et al. 2019). The alignment was manually adjusted using AliView version 1.27 (Larsson 2014). Sequences of *Helicobasidiummompa* and *Pachnocybeferruginea* Berk. obtained from GenBank were used as an outgroup to root trees following Henk and Vilgalys (2007) in the ITS analysis (Fig. 1).

Maximum parsimony (MP), maximum likelihood (ML) and Bayesian Inference (BI) analyses were applied to the three combined datasets. The phylogenetic analysis method was adopted by previous study (Zhao and Wu 2017; Yang et al. 2023; Dong et al. 2024). MP analysis was performed in PAUP* version 4.0b10 (Swofford 2002). All of the characteristics were equally weighted and gaps were treated as missing data. Trees were inferred using the heuristic search option with TBR branch swapping and 1,000 random sequence additions. Max-trees were set to 5,000, branches of zero length were collapsed and all most-parsimonious trees were saved. Clade robustness was assessed using bootstrap (BT) analysis with 1,000 replicates (Felsenstein 1985). Descriptive tree statistics tree length (TL), the consistency index (CI), the retention index (RI), the rescaled consistency index (RC) and the homoplasy index (HI) were calculated for each most-parsimonious tree generated. ML was inferred using RAxML-HPC2 through the CIPRES Science Gateway (Miller et al. 2012). Branch support (BS) for ML analysis was determined using 1,000 bootstrap replicates and evaluated under the gamma model.

MrModeltest 2.3 (Posada and Crandall 1998; Nylander 2004) was used to determine the best-fit evolution model for each data set for Bayesian inference (BI). Bayesian inference was calculated with MrBayes3.1.2 with a general time reversible (GTR+G+I) model of DNA substitution and a gamma distribution rate variation across sites (Ronquist et al. 2012). Four Markov chains were run for 2 runs from random starting trees for 2.9 million generations (Fig. 1) and trees were sampled every 100 generations. The first one-fourth generations were discarded as burn-in. A majority rule consensus tree of all remaining trees was calculated. Branches were considered as significantly supported if they received maximum likelihood bootstrap (ML) ≥ 70%, maximum parsimony bootstrap (MP) ≥ 50%, or Bayesian posterior probabilities (PP) ≥ 0.95.

### ﻿Pairwise homoplasy test

The Genealogical concordance phylogenetic species recognition analysis (GCPSR) is a tool used to check significant recombinant events. The data were analysed using SplitsTree 4 with the pairwise homoplasy Фw, PHI test to determine the recombination level within closely related species (Bruen et al. 2006; Huson and Bryan 2006; Quaedvlieg et al. 2014). One-locus dataset (ITS) with closely related species were used for the analyses. PHI results lower than 0.05 (Φw < 0.05) indicates a significant recombination is present in the dataset. The relationships between closely related taxa were visualised by constructing split graphs from the concatenated datasets, using the LogDet transformation and splits decomposition options.

## ﻿Result

### ﻿Molecular phylogeny

The dataset based on ITS (Fig. 1) comprises sequences from 43 fungal specimens representing 35 species. The alignment length of this dataset is 474 characters, of which 267 characters are constant, 45 characters are variable with no information and 162 characters have no information. Maximum parsimony analysis yielded three equally parsimonious trees (TL = 967, CI = 0.3516, HI = 0.6484, RI = 0.4543, RC = 0.1597). Bayesian analysis and ML analysis resulted in a similar topology as MP analysis with an average standard deviation of split frequencies of 0.007729 (BI) and the effective sample size (ESS) across the two runs is double the average ESS (avg. ESS) = 861.

The phylogenetic tree (Fig. 1), inferred from the ITS sequences, highlighted that *Septobasidiummacrobasidium* was clustered with *S.maesae* C.X. Lu & L. Guo. Furthermore, *S.puerense* was retrieved as a sister to *S.carestianum*. The new species *S.wuliangshanense* was the sister to *S.aquilariae* C.L. Zhao with strong supports.

**Figure 2.**
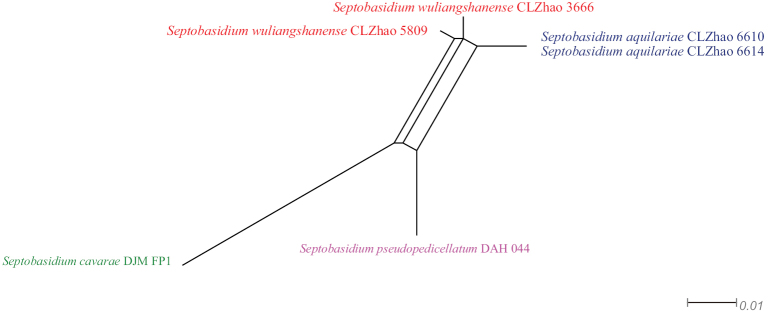
Split graphs showing the results of PHI test for the ITS data of *Septobasidiumwuliangshanense* and closely related taxa using LogDet transformation and splits decomposition. PHI test results Φw ≤ 0.05 indicate that there is significant recombination within the dataset. New taxa are in red while closely related species to new species are in other colours.

Application of PHI test to the ITS tree-locus sequences revealed no recombination level within phylogenetically related species. The test results of ITS sequence dataset show Φw = 0.5697 (Φw > 0.05) no recombination is present in the new species *Septobasidiumwuliangshanense* with *S.aquilariae*, *S.cavarae* Bres. and *S.pseudopedicellatum* Burt (Fig. 2).

### ﻿Taxonomy

#### ﻿ Septobasidium macrobasidium

Taxon classificationFungiSeptobasidialesSeptobasidiaceae

Q.Q. Jiang & C.L. Zhao sp. nov.

2BB48D40-A80B-5FC3-A09B-08CB6B30422C

853680

Figs 3 , 4

##### Holotype.

China • Yunnan Province, Pu’er, Jingdong County, Wuliangshan National Nature Reserve, 24°29'17"N, 100°40'27"E, altitude: 1800 m a.s.l., on the living tree of angiosperm, leg. C.L. Zhao, 6 January 2019, CLZhao 9624 (SWFC).

##### Diagnosis.

Differs from other *Septobasidium* species by the coriaceous basidiomata with a cream surface, cylindrical basidia (48.5–83 × 6.5–13 µm), and thin-walled, narrowly cylindrical basidiospores with septa, measuring as 7–9 × 3.5–4.5 µm.

**Figure 3.**
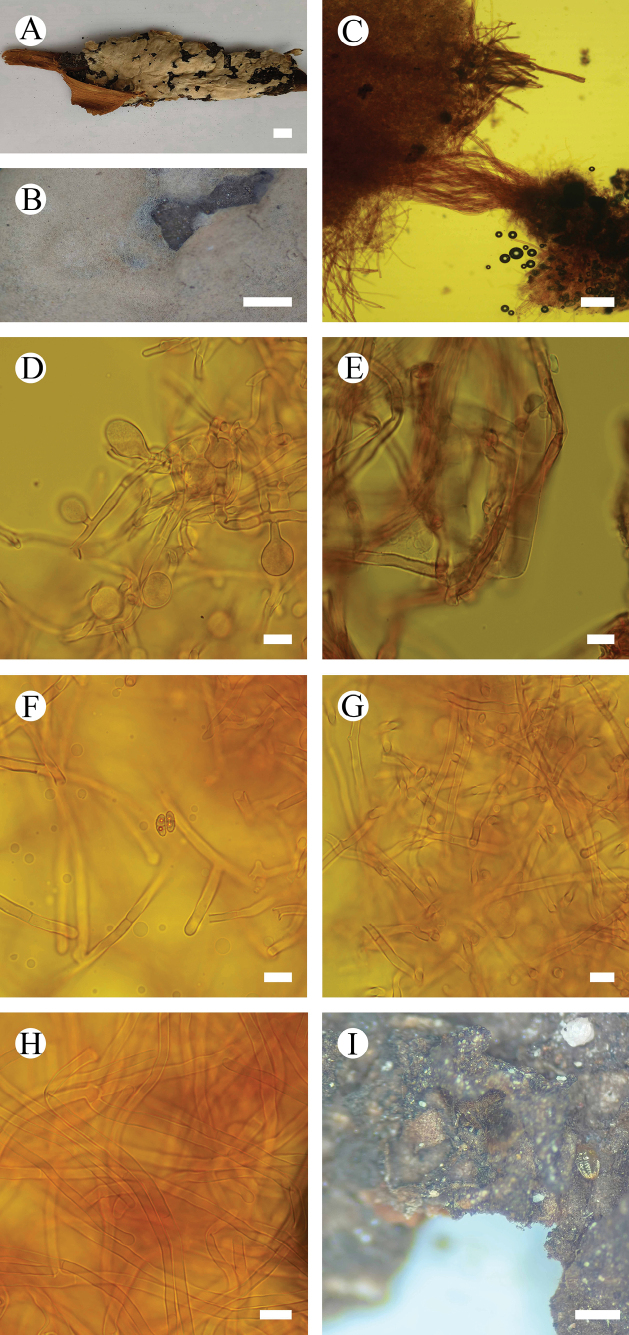
*Septobasidiummacrobasidium* (holotype, CLZhao 9624) **A, B** basidiomata on branch **C** sections of basidiomata **D** probasidia **E** basidia **F** basidiospore **G** haustoria **H** hyphae **I** scale insect on branches. Scale bars: 1 cm (**A**); 1 mm (**B**); 100 µm (**C**); 10 µm (**D–H**); 1 mm (**I**).

##### Etymology.

*Macrobasidium* (Lat.): refers to the larger basidia of the type specimen.

##### Description.

Basidiomata perennial, resupinate, easy to separate from substrate, coriaceous upon drying, up to 9 cm long, 5 cm wide, 2 mm thick. Hymenial surface smooth, slightly cream when fresh, cream upon drying. Sterile margin narrow, white to cream, up to 0.5 mm.

Hyphal system monomitic, generative hyphae with simple septa, pale brown, thick-walled, frequently branched, interwoven. In section 850–1850 µm thick; subiculum pale brown, 30–80 µm thick; pillars brown, 350–550 µm high, 85–200 µm wide, hyphae of pillars 2–5 µm thick, hyaline or brown, forming 2–3 horizontal layers.

**Figure 4.**
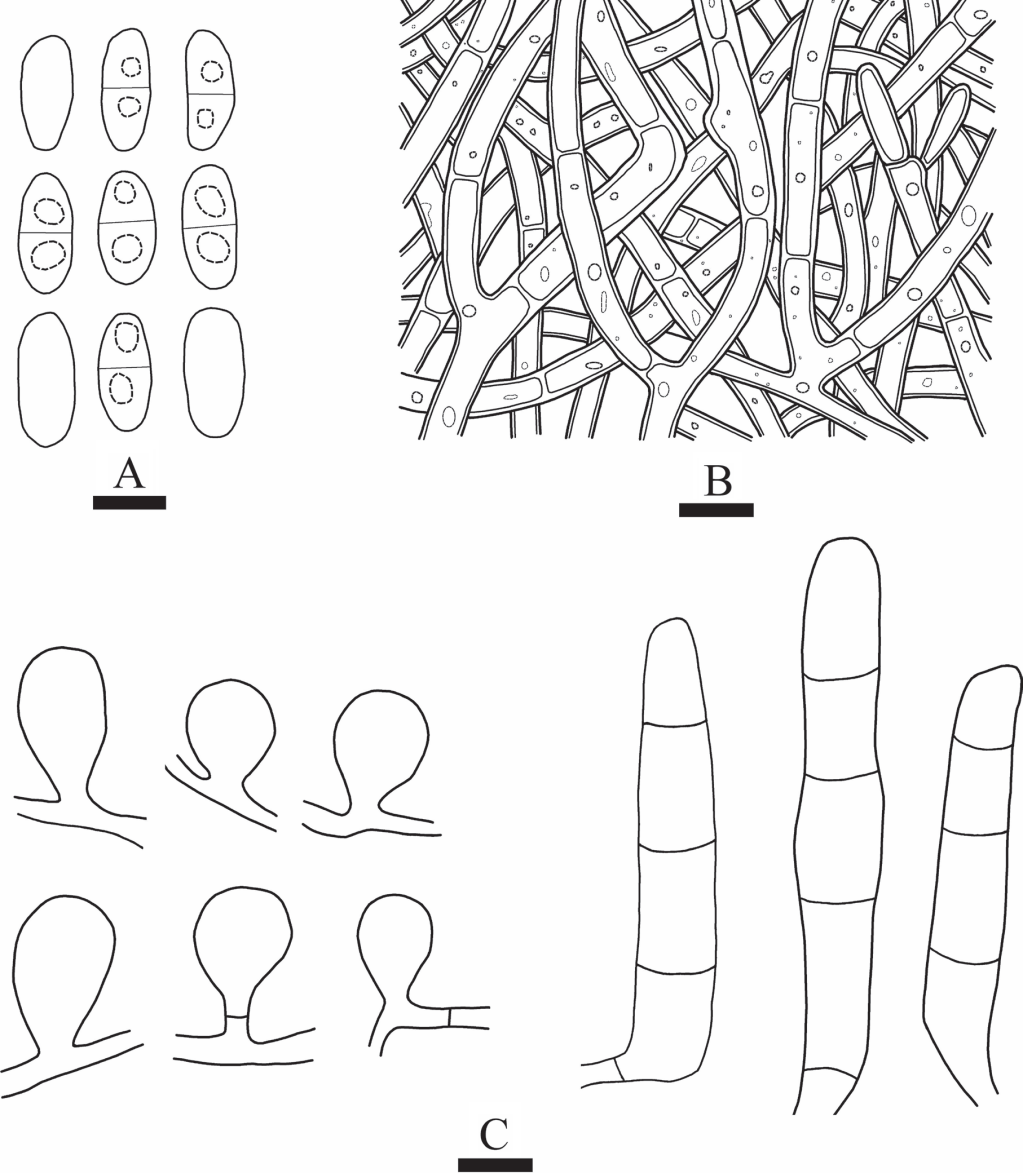
Microscopic structures of *Septobasidiummacrobasidium* (holotype, SWFC 9624) **A** basidiospores **B** generative hyphae from hyphal layer **C** probasidia and basidia. Scale bars: 5 µm (**A**); 10 µm (**B, C**).

Basidia arising directly from the generative hyphae, basidia cylindrical, straight, 4-celled, 48.5–83 × 6.5–13 µm, colourless. Probasidia subglobose or ovoid, 13–23 × 7–20.5 µm, colourless, persistent. Haustoria consisting of irregularly coiled hyphae.

Basidiospores narrowly cylindrical, colourless, thin-walled, with septa, (6.5–)7–9(–9.5) × (3–)3.5–4.5(–5) µm, L = 8.09 µm, W = 3.94 µm, Q = 1.55–2.83 (n = 60/2).

##### Habitat and distribution.

Growing on the plant Betulaceae Gray, associated with the insect of Diaspididae Ferris.

##### Additional specimen examined

**(*paratype*).** China • Yunnan Province, Pu’er, Jingdong County, Wuliangshan National Nature Reserve, 24°29'17"N, 100°40'27"E, altitude: 1800 m a.s.l., on the living tree of angiosperm, leg. C.L. Zhao, 6 January 2019, CLZhao 9658 (SWFC).

#### ﻿ Septobasidium puerense

Taxon classificationFungiSeptobasidialesSeptobasidiaceae

Q.Q. Jiang & C.L. Zhao sp. nov.

67C84587-4068-5C91-9DFE-1B62241520A3

853681

Figs 5 , 6

##### Holotype.

China • Yunnan Province, Pu’er, Jingdong County, The Forest of Pineapple, 24°30'58"N, 100°52'31"E, altitude: 2000 m a.s.l., on the living tree of angiosperm, leg. C.L. Zhao, 4 January 2019, CLZhao 9430 (SWFC).

##### Diagnosis.

Differs from other *Septobasidium* species by a cinnamon brown to chestnut brown surface, subglobose to pyriform probasidia (10.5–19.5 × 5.5–9 µm), and two types of haustoria consisting of irregularly coiled hyphae and spindle-shape.

##### Etymology.

*Puerense* (Lat.): refers to the locality (Pu’er) of the type specimen.

##### Description.

Basidiomata perennial, resupinate, hard to separate from substrate, coriaceous upon drying, up to 15 cm long, 1 cm wide,1 mm thick. Hymenial surface smooth, pale brown when fresh, cinnamon brown to chestnut brown upon drying. Sterile margin slightly brown, up to 1 mm.

**Figure 5.**
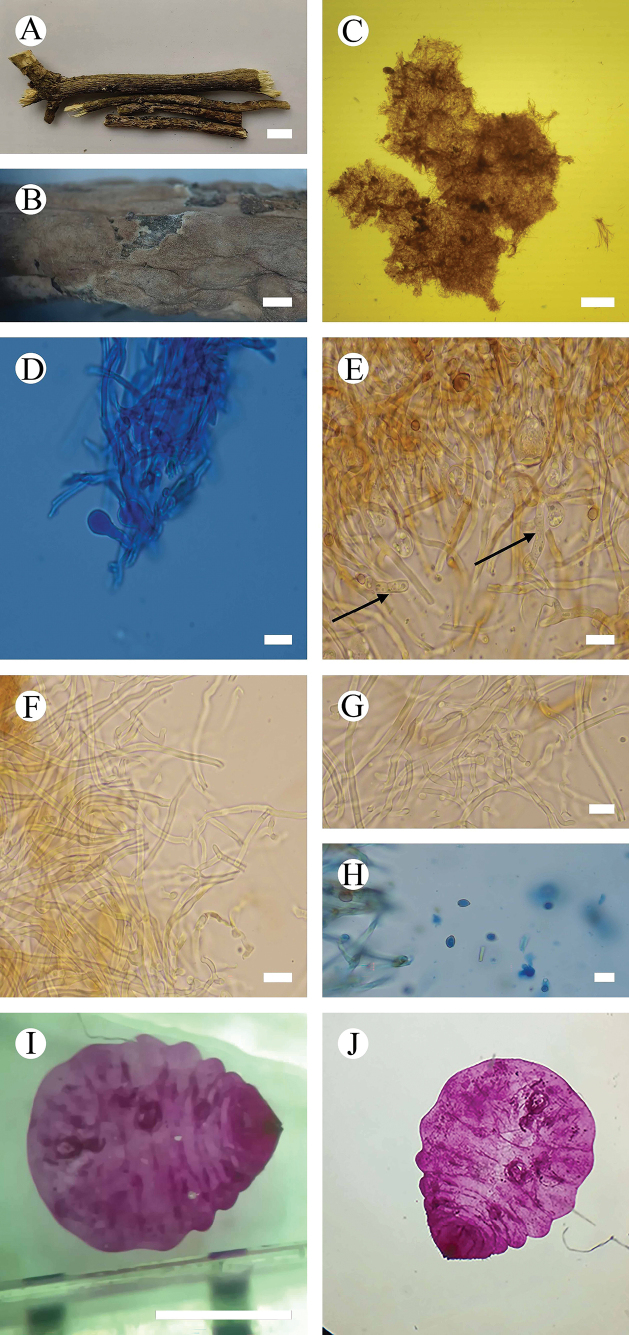
*Septobasidiumpuerense* (holotype, CLZhao 9430) **A, B** basidiomata on branch **C** sections of basidiomata **D** probasidia **E** basidia (arrow) **F** hyphae **G** haustoria consisting of irregularly coiled hyphae **H** the spindle-shaped haustoria **I, J** scale insect on branches. Scale bars: 1 cm (**A**); 1 mm (**B**); 100 µm (**C**); 10 µm (**D–H**); 1 µm (**I**).

Hyphal system monomitic, generative hyphae with simple septa, pale brown, thick-walled. In section 380–650 µm thick; subiculum pale brown, 10–30 µm thick; pillars brown, 170–380 µm high, 40–85 µm wide, hyphae of pillars 1.5–3.5 µm thick, colorless, with closely packed parallel upright threads, forming 2–3 horizontal layers.

Basidia arising directly from the generative hyphae, cylindrical or slightly irregular, slightly curved, 2-3-celled, 17–30.5 × 3–6.5 µm, colourless. Probasidia subglobose to pyriform, 10.5–19.5 × 5.5–9 µm, colorless, probasidia cell persistent after the formation of the basidia. Basidiospores not seen. Haustoria with two types: 1) consisting of irregularly coiled hyphae; 2) spindle-shape.

**Figure 6.**
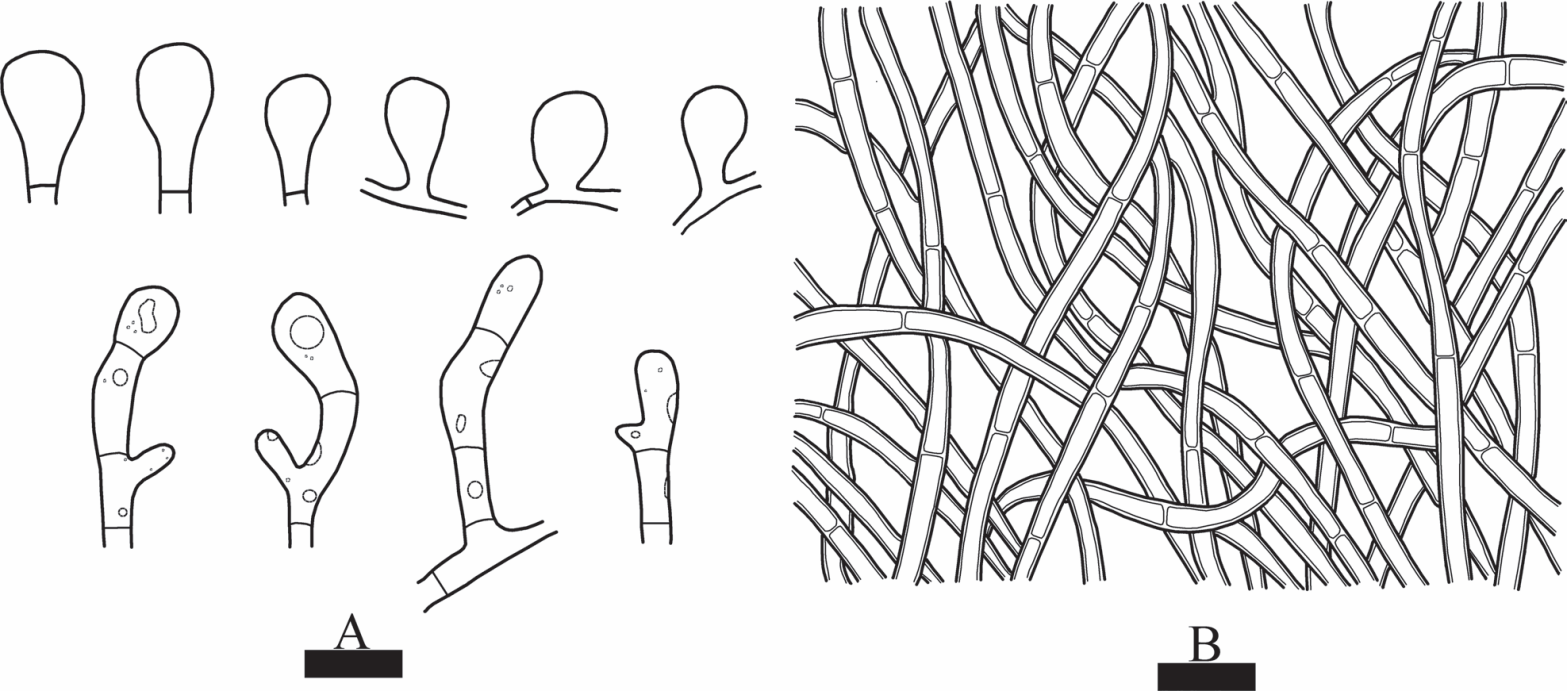
Microscopic structures of *Septobasidiumpuerense* (holotype, CLZhao 9430) **A** probasidia and basidia **B** generative hyphae from hyphal layer. Scale bars: 10 µm (**A, B**).

##### Habitat and distribution.

Growing on the plant Berberidaceae Juss, associated with the insect species *Pseudaulacaspispentagona* (Targioni Tozzetti).

##### Additional specimen examined

**(*paratype*).** China • Yunnan Province, Pu’er, Jingdong Country, Wuliangshan National Nature Reserve, 24°29'17"N, 100°40'27"E, altitude: 1800 m a.s.l., on the living tree of angiosperm, leg. C.L. Zhao, 5 October 2017, CLZhao 4298 (SWFC).

#### ﻿ Septobasidium wuliangshanense

Taxon classificationFungiSeptobasidialesSeptobasidiaceae

Q.Q. Jiang & C.L. Zhao sp. nov.

24DB22B9-7D95-5F70-BE6F-C7FDF88C8F6E

853679

Figs 7 , 8

##### Holotype.

China • Yunnan Province, Pu’er, Zhenyuan County, Heping Town, Liangzi Village, Wuliangshan National Nature Reserve, 24°29'17"N, 100°40'27"E, altitude: 1860 m a.s.l., on the living tree of angiosperm, leg. C.L. Zhao, 15 January 2018, CLZhao 5809 (SWFC).

##### Diagnosis.

Differs from other *Septobasidium* species by the coriaceous basidiomata with a slightly brown surface, cylindrical or slightly irregular basidia, pyriform to subglobose or ovoid probasidia (7.5–13 × 4.5–9 µm), and the haustoria consisting of irregularly coiled hyphae.

##### Etymology.

*Wuliangshanense* (Lat.): refers to the locality (Wuliangshan) of the type specimen.

##### Description.

Basidiomata perennial, resupinate, easy to separate from substrate, coriaceous upon drying, up to 10 cm long, 2 cm wide, 1 mm thick. Hymenial surface smooth, cream to pale brown when fresh, slightly brown upon drying. Sterile margin cream to slightly brown, up to 2 mm.

**Figure 7.**
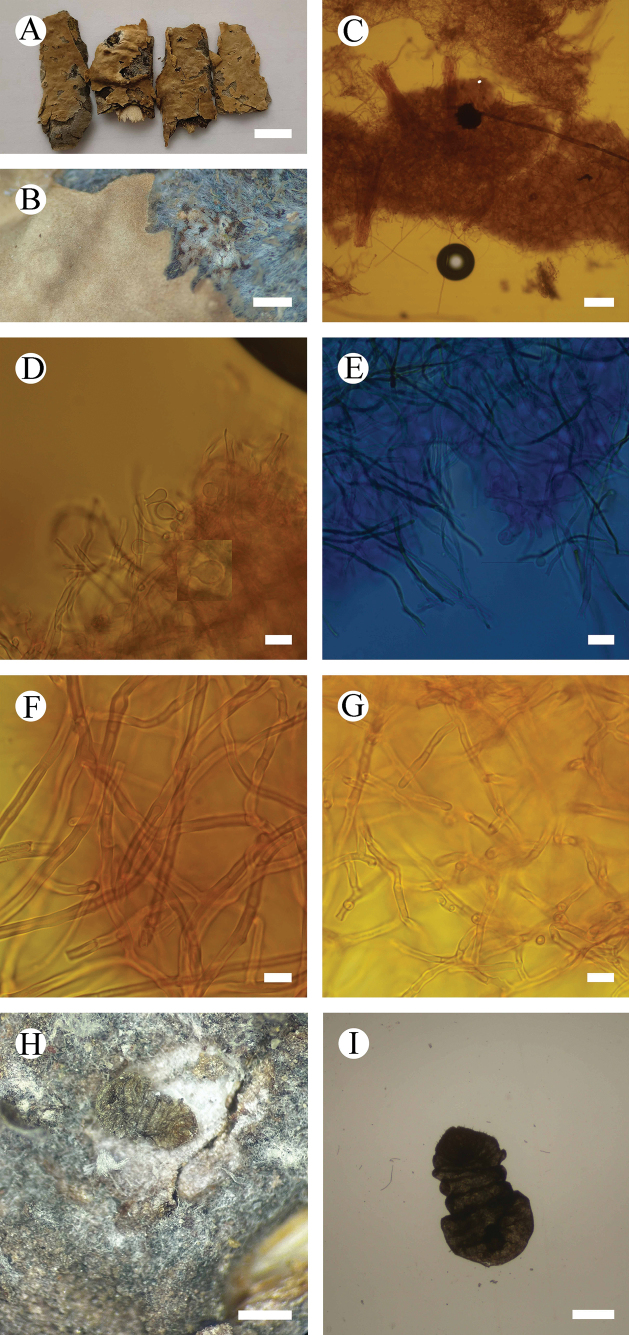
*Septobasidiumwuliangshanense* (holotype, CLZhao 5809) **A, B** basidiomata on branch **C** sections of basidiomata **D** probasidia (arrow) **E** basidium (arrow) **F** hyphae **G** haustoria **H, I** scale insect on branches. Scale bars: 1 cm (**A**); 1 mm (**B**); 100 µm (**C**); 10 µm (**D–G**); 1 mm (**H**); 10 mm (**I**).

Hyphal system monomitic, generative hyphae with simple septa, pale brown, thick-walled. In section 660–1200 µm thick; subiculum pale brown, 20–50 µm thick; pillars brown, 150–300 µm high, 30–150 µm wide, hyphae of pillars 3–4 µm thick, brown, forming 2–3 horizontal layers.

Basidia arising directly from the generative hyphae, cylindrical or slightly irregular, colourless, straight or slightly curved, 2-3-celled, 21.5–29 × 5.5–9 µm. Probasidia pyriform, subglobose or ovoid, 7.5–13 × 4.5–9 µm, colorless, probasidia cell persistent after the formation of the basidia. Haustoria consisting of irregularly coiled hyphae. Basidiospores not seen.

**Figure 8.**
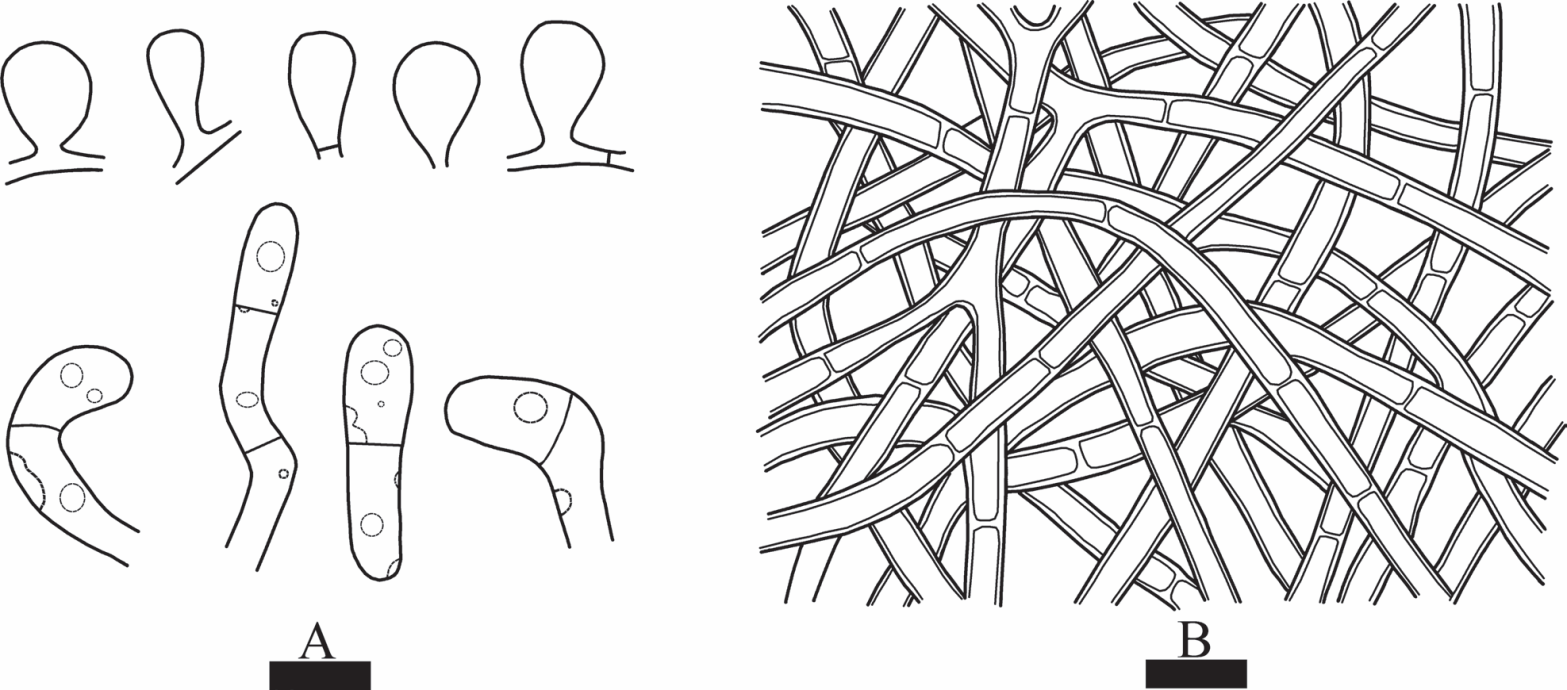
Microscopic structures of *Septobasidiumwuliangshanense* (holotype, CLZhao 5809) **A** probasidium and basidia **B** generative hyphae from hyphal layer. Scale bars: 10 µm (**A, B**).

##### Habitat and distribution.

Growing on the plant Fagaceae Dumort, associated with the insect genus *Aulacaspis* Cockerell.

##### Additional specimen examined

**(*paratype*).** China • Yunnan Province, Pu’er, Jingdong County, Wuliangshan National Nature Reserve, in association with the genus *Aulacaspis* on Rosaceae, 24°29'17"N, 100°40'27"E, altitude: 1800 m a.s.l., on the living tree of angiosperm, leg. C.L. Zhao, 2 October 2017, CLZhao 3666 (SWFC).

## ﻿Discussion

Many recently described wood-inhabiting fungal taxa have been reported worldwide, including in the genus *Septobasidium* (Patouillard 1892; Bresadola and Saccardo 1897; Burt 1916; Lloyd 1919; Couch 1929, 1935, 1938, 1946; Yamamoto 1956; Gómez and Henk 2004; Henk 2005; Chen and Guo 2009a, 2009b, 2010, 2011a, 2011b, 2011c, 2012; Lu and Guo 2009a, 2009b, 2010a, [Bibr B40][Bibr B41], 2013, 2014; Lu et al. 2010). The diversity of *Septobasidium* is rich in China. Prior to this study, the following 58 *Septobasidium* species were reported from China, especially in subtropics and tropics (Lu and Guo 2009a, 2009b, 2010a, 2010b, 2011; Lu et al. 2010; Chen and Guo 2011a, 2011b, 2011c, 2012; Li and Guo 2013, 2014; Ma et al. 2019). Several *Septobasidium* species have been described from Yunnan Province (Lu and Guo 2010b, 2011; Ma et al. 2019). In the present study, three new species, *S.macrobasidium*, *S.puerense* and *S.wuliangshanense* are described based on phylogenetic analyses and morphological characteristics. In addition, the PHI test (Fig. 2) was carried out to confirm that there is no recombination present in the new species *S.wuliangshanense* compared with closely related taxa.

Based on ITS topology (Fig. 1), the phylogenetic tree includes the type species *Septobasidiumvelutinum*, which is collected from Costa Rica and most species of this genus have persistent probasidia, except for *S.aquilariae*, *S.gomezii* Henk, *S.hainanense* C.X. Lu & L. Guo, *S.pallidum* Couch, *S.septobasidioides* (Henn.) Höhn. & Litsch. and *S.westonii* Couch. Most *Septobasidium* species have irregularly coiled haustoria and the spindle-shaped haustoria of *S.fumigatum* Burt, *S.griseum* Couch, *S.grandisporum* Couch, *S.pilosum* Boedijn & B.A. Steinm and *S.sinuosum* Couch. However, *S.puerense* has two types of haustoria: 1) consisting of irregularly coiled hyphae; 2) spindle-shape. Most *Septobasidium* species have pillars, except for *S.arachnoideum* (Berk. & Broome) Bres., *S.burtii* Lloyd, *S.canescens* Burt, *S.cavarae*, *S.fumigatum*, *S.grandisporum*, *S.michelianum* (Caldesi) Pat., *S.pilosum*, *S.pinicola* Snell, *S.sinuosum*, *S.taxodii* Couch and *S.wilsonianum* L.D. Gómez & Kisim.-Hor.

Phylogenetically, based on the ITS topology (Fig. 1), *Septobasidiummacrobasidium* is clustered with *S.maesae*. The new species *S.puerense* is closely related to *S.carestianum* and *S.wuliangshanense* is sister to *S.aquilariae* with strong supports. But morphologically *S.maesae* differs from *S.macrobasidium* by its greyish-brown hymenial surface and smaller basidia (28–55 × 7.5–11.5 µm), brown, fusiform basidiospore without septa (18–19.5 × 4–5 µm; Lu and Guo 2009a). The species *S.carestianum* differs from *S.puerense* by its iuventute avellaneas deinde cinnamomeas, superficiei sub lente pruinosula hymenial surface and the larger sphaerica probasidia (15.1–11.3 µm) and 4-celled, apicem acuta, longer cylindrica basidia in senectute brunnea (62–71 × 5–6.7 µm), ellipticae and flexae sporae (21–23 × 4.2–5 µm; Gómez and Henk 2004). The species *S.aquilariae* differs from *S.wuliangshanense* by its smaller basidia (15–26.5 × 4–6 µm) and without a probasidia cell and reniform basidiospores (11–19 × 4–7.5 µm), habitat and distribution growing in association with *Pseudaulacaspis* sp. on *Aquilariasinensis* (Lour.) Spreng. (Ma et al. 2019). Further, application of PHI test to the ITS tree-locus sequences revealed no recombination level within phylogenetically in these two species (Fig. 2).

Morphologically, *Septobasidiumcokeri* Couch. differs from *S.macrobasidium* by its pure white hymenial surface and restricted growth on *Quercusrubra* L. (Gómez and Henk 2004). The species *S.hainanense* differs in its purple hymenial surface and smaller basidia (25–36 × 7–13 µm; Lu and Guo 2010a). The taxon *S.maesae* differs by its perennial basidiomata peeled off after maturity and smaller basidia (28–55 × 7.5–11.5 µm; Lu and Guo 2009a).

*Septobasidiumguangxiense* Wei Li bis & L. Guo differs from *S.puerense* in its yellowish brown hymenial surface with numerous fissures at maturity and larger basidia (27–38 × 5–10 µm; Li and Guo 2014). The species *S.hoveniae* Wei Li bis, S.Z. Chen, L. Guo & Yao Q. Ye differs in its cinnamon-brown hymenial surface and growth on *Hoveniaacerba* (Li and Guo 2013; *S.polygoni* C.X. Lu & L. Guo differs in its white to cinnamon-brown hymenial surface and growth on *Polygonumcampanulatum* Hook. f. (Lu and Guo 2010b); *S.reevesiae* S.Z. Chen & L. Guo differs in its thicker section (1.65–2.20 mm) and larger basidia (37–55 × 8–13 µm) and growth on *Reevesialongipetiolata* Merr. & Chun (Chen and Guo 2012) and *S.transversum* Wei Li bis & L. Guo differs in its cinnamon-brown basidiomata, its transverse layer at the pillar bases and larger basidia (42–60 × 9–16 µm; Li and Guo 2014).

Morphologically, several species of *Septobasidiumbroussonetiae* C.X. Lu, L. Guo & J.G. Wei, *S.brunneum* Wei Li bis & L. Guo, *S.capparis* S.Z. Chen & L. Guo, *S.euryae-groffii* C.X. Lu & L. Guo, *S.fissuratum* Wei Li bis & L. Guo and *S.gaoligongense* C.X. Lu & L. Guo are similar to *S.wuliangshanense* were found in China. However, *S.broussonetiae* is distinguished by its cracking basidiomata and growth on *Broussonetiapapyrifera* (L.) L’Hér. ex Vent. (Lu et al. 2010); *S.brunneum* differs in its purple-brown hymenial surface with many cracks and growth on *Eurya* sp. (Li and Guo 2014); *S.capparis* differs by its thicker section (less than 2 mm thick) and larger basidia (45–56 × 8–12 µm; Chen and Guo 2012); *S.euryae-groffii* is distinguished by its cinnamon to chestnut brown hymenial surface and growth on *Euryagroffii* (Lu and Guo 2010b); *S.fissuratum* differs in its larger basidia (32–45 × 6–9 µm) and growth on *Castanea* sp. (Li and Guo 2013); *S.gaoligongense* differs in its dark brown hymenial surface and thinner section (260–580 µm; Lu and Guo 2010b).

Based on our phylogenetic and morphological research results, 61 species have been reported from China, including newly described in the present study and other recently published papers in this country (Gómez and Henk 2004; Lu and Guo 2009a, 2009b, 2010a, 2010b, 2011; Lu et al. 2010; Chen and Guo 2011a, 2011b, 2011c, 2012; Li and Guo 2013, 2014; Ma et al. 2019). It seems that the species diversity of *Septobasidium* is rich in China. Although the taxa of *Septobasidium* are well studied in the present paper and the species diversity, taxonomy and phylogeny of *Septobasidium* and related genera are still unresolved. A comprehensive study on this issue is urgently needed.

## Supplementary Material

XML Treatment for Septobasidium macrobasidium

XML Treatment for Septobasidium puerense

XML Treatment for Septobasidium wuliangshanense

## ﻿Additional information

### Author ORCIDs

Qianquan Jiang https://orcid.org/0009-0003-0644-3638

Zhengli Kang https://orcid.org/0009-0003-1878-6822

Xubo Wang https://orcid.org/0000-0001-5996-6027

Changlin Zhao https://orcid.org/0000-0002-8668-1075

### Data availability

All of the data that support the findings of this study are available in the main text.
